# Exercise and Fitness Neuroprotective Effects: Molecular, Brain Volume and Psychological Correlates and Their Mediating Role in Healthy Late-Middle-Aged Women and Men

**DOI:** 10.3389/fnagi.2021.615247

**Published:** 2021-03-08

**Authors:** Alba Castells-Sánchez, Francesca Roig-Coll, Rosalia Dacosta-Aguayo, Noemí Lamonja-Vicente, Angelika K. Sawicka, Pere Torán-Monserrat, Guillem Pera, Pilar Montero-Alía, Antonio Heras-Tebar, Sira Domènech, Marc Via, Kirk I. Erickson, Maria Mataró

**Affiliations:** ^1^Departament of Clinical Psychology and Psychobiology, University of Barcelona, Barcelona, Spain; ^2^Institut de Neurociències, University of Barcelona, Barcelona, Spain; ^3^Institut de Recerca Pediàtrica Hospital Sant Joan de Déu, Esplugues de Llobregat, Spain; ^4^Applied Cognitive Neuroscience Lab, Department of Human Physiology, Medical University of Gdansk, Gdansk, Poland; ^5^Unitat de Suport a la Recerca Metropolitana Nord, Institut Universitari d'Investigació en Atenció Primària Jordi Gol (IDIAP Jordi Gol), Mataró, Spain; ^6^Institut de Diagnòstic per la Imatge, Hospital Universitari Germans Trias i Pujol, Barcelona, Spain; ^7^Department of Psychology, University of Pittsburgh, Pittsburgh, PA, United States

**Keywords:** exercise, fitness, molecular biomarkers, brain volume, psychological health, cognition

## Abstract

**Background:** Although exercise is known to have a neuroprotective effect in aging, the mediators underlying the exercise-cognition association remain poorly understood. In this paper we aimed to study the molecular, brain, and behavioral changes related to physical activity and their potential role as mediators.

**Methods:** We obtained demographic, physical activity outcomes [sportive physical activity and cardiorespiratory fitness (CRF)], plasma biomarkers (TNF-α, ICAM-1, HGF, SDF1-α, and BDNF), structural-MRI (brain volume areas), psychological and sleep health (mood, depressive and distress symptoms, and sleep quality), and multi-domain cognitive data from 115 adults aged 50–70 years. We conducted linear regression models and mediation analyses stratifying results by sex in a final sample of 104 individuals [65 women (age = 56.75 ± 4.96) and 39 men (age = 58.59 ± 5.86)].

**Results:** Women engaging in greater amounts of exercising showed lower TNF-α levels and greater dorsolateral prefrontal cortex and temporal lobe volumes. Men engaging in greater amounts of exercise showed greater temporal lobe volumes. CRF levels were not related to any of the analyzed outcomes in women but in men higher CRF was associated with lower TNF-α, HGF and ventricle volumes, greater volume of temporal and parietal lobes and fewer depressive symptoms and better mood. In men, reduced TNF-α and HGF levels mediated brain and cognitive CRF-related benefits.

**Conclusion:** Our results show that exercise is a promising approach for influencing inflammation and brain volume and also contributes to ongoing discussions about the physiological mediators for the association between CRF and cognition in men.

## Introduction

“Let's move your body; let's rock your cells, brain and self!” Epidemiological studies have indicated that an active lifestyle including exercise positively impacts several major hallmarks of aging and has neuroprotective benefits (Garatachea et al., 2015). Exercise, a subtype of physical activity (PA) applied in a regular manner in order to improve physical fitness, is related to reduced risk of dementia and better cognitive health in clinical and non-clinical populations, with greatest effects for measures of executive function (Barha et al., 2017; Northey et al., 2018). Those benefits have been described when using a measure of self-reported exercise habits and when assessing physiological correlates of habitual PA such as cardiorespiratory fitness (CRF). CRF is the ability of the cardiovascular system to supply oxygen to the organism during sustained PA. Previous literature has identified potential mechanisms of the exercise-cognition association at multiple levels: molecular, brain, and behavioral. In this paper we will discuss them as Level 1, 2, and 3 mechanisms, respectively, based on Stillman et al. (2016).

At the molecular level, Level 1, aging is related to altered levels of inflammatory, oxidative stress, metabolic and neuronal, and cell growth markers (Garatachea et al., 2015). Exercise reduces levels of systemic inflammation modulating markers such as C-reactive protein (CRP), Interleukin 6 (IL-6), Interleukin 1 (IL-1), and Tumor Necrosis Factor alpha (TNF-α) (Sallam and Laher, 2016). However, Woods et al. (2012) indicated that the evidence for a relationship between CRF and TNF-α levels in humans was still insufficient for making definitive conclusions. Studies with mice have demonstrated that exercise also produces an increased shear stress on endothelial cells that modulates other markers of the inflammatory process such as Intercellular Adhesion Molecule 1(ICAM-1), Nuclear factor-κB (NF-κB), Mitogen-activated protein kinase (MAPK), and Cyclooxygenases (COX-2) (Sallam and Laher, 2016). Shear stress and mechanical load related to exercise induce changes in other anabolic and metabolic growth factors that affect muscles and bones such as Hepatocyte growth factor (HGF), Vascular endothelial grow factor (VEGF), and Insulin-like growth factor-1 (IGF1). HGF, which is commonly related to obesity and insulin resistance and is capable of modulating the inflammatory response, promotes angiogenesis, and neuroprotection in the brain (Kiliaan et al., 2014). Exercise-induced VEGF is also related to angiogenesis and improved circulation in humans. It upregulates the chemokine Stromal cell-derived factor 1 (SDF1-α), also known CXCL12 (Stimpson et al., 2018), which promotes both endothelial progenitor cells and the endothelial nitric oxide synthase enzyme in mice (Gertz et al., 2006). Exercise also influences brain derived neurotrophic factor (BDNF), a neuronal growth factor involved in neurogenesis. A systematic review (Huang et al., 2014) reported a negative association between long-term regular PA and CRF with peripheral BDNF in observational studies. Those results may reflect a more efficient uptake mechanism of circulating BDNF into the brain in active subjects (Currie et al., 2009).

At a more macroscopic level, Level 2, reviews and systematic reviews reported beneficial effects of exercise on brain volume in healthy older adults, specifically for areas more related to normal brain aging (Erickson et al., 2014; Sexton et al., 2016; Stillman et al., 2016). Cross-sectional studies showed that higher amounts of PA were associated with greater total brain volume (Benedict et al., 2013; Spartano et al., 2019) and gray matter (GM) volume in the frontal lobe (Erickson et al., 2010; Flöel et al., 2010; Bugg and Head, 2011; Eyme et al., 2019), hippocampus (Erickson et al., 2010; Yamamoto et al., 2017; Raichlen et al., 2019), cingulate cortex (Flöel et al., 2010), precuneus (Benedict et al., 2013; Eyme et al., 2019), and nucleus accumbens (Yamamoto et al., 2017). CRF levels have also been positively correlated with overall gray matter (Raichlen et al., 2019), multiple areas of the frontal lobe (Gordon et al., 2008; Weinstein et al., 2012; Wittfeld et al., 2020), medial-temporal lobe (Gordon et al., 2008; Wittfeld et al., 2020), hippocampus (Erickson et al., 2009; Szabo et al., 2011; Wittfeld et al., 2020), and cingulate cortex (Wittfeld et al., 2020) volume in healthy older adults. Findings related to white matter (WM) indicated that engaging more frequently in PA could increase WM global volume (Gow et al., 2012; Benedict et al., 2013; Arnardottir et al., 2016). Only a few studies have examined the relationship between CRF and WM volume and did not find significant results (Burns et al., 2008; Gordon et al., 2008; Honea et al., 2009).

At a behavioral level, Level 3, aging is commonly associated with increased sedentarism (Copeland et al., 2015), changes in sleeping patterns (Li et al., 2018), and more psychological problems such as depression or anxiety symptoms (World Health Organization (WHO), 2017). For older adults, exercising is related with better physical and mental health (Bertheussen et al., 2011), quality of life (Fox et al., 2007), and well-being (Lee and Hung, 2011; Black et al., 2015). Higher levels of PA (Strawbridge et al., 2002) and CRF (Sui et al., 2009; Willis et al., 2018) are also protective for prevalent and incident depression. PA is also associated with better sleep quality (Kline et al., 2013; Tan et al., 2018) and efficiency (Kline et al., 2013; Wilckens et al., 2018) and total sleep time (Murray et al., 2017). However, the relationship between sleep patterns and CRF in healthy older adults is not well-established.

Conceiving mechanisms at multiple levels might be helpful to better understand the relationship between exercise and cognition. As the evidence suggests, exercise might initiate a molecular cascade that promotes macroscopic changes in the brain and/or behaviors that in turn enhance cognition. For example, PA has been related to reduced inflammatory profile and greater brain volume (Stillman et al., 2016). However, there is a poor understanding of the mediating role of these variables in the PA-cognition relationship and the multiple pathways by which microscopic and macroscopic biomarkers might influence each other to promote cognition are in current research. Identifying these pathways by which the benefits of exercise are realized is a challenge not only due to the multi-level mechanisms but also because of the role of factors that may moderate the association such as sex (Barha et al., 2019). Previous evidence suggests that both women and men might positively benefit cognition from exercise, although the CRF-cognition relationship was only significant in men (Castells-Sánchez et al., 2020). Moreover, there are sex differences in the mediators of this relationship described in the ongoing research. For example, greater PA was related to reduced TNF-α only in men (Elosua et al., 2005), while daily walking has been associated with greater hippocampal (Varma et al., 2015) and dorsolateral prefrontal volume (Barha et al., 2020) and larger surfaces of the subiculum (Varma et al., 2016) in women, but not in men. Therefore, addressing the role of these mechanisms stratified by sex might be interesting to better understand exercise as a personalized approach to enhance cognitive health.

To our knowledge, previous research focused on single levels of analyses when examining and describing possible mechanisms of exercise on cognition (Stillman et al., 2016). In this paper, we first aim to study the relationship between exercise and CRF with key markers at molecular, brain volume, and behavioral levels of analysis and further stratifying the results by sex because of established sex-related differences in many of these measures. Secondly, we aim to study their potential mediating role of each of these markers in the exercise-cognition relationship and perform exploratory analyses to address the potential pathways by which these markers might influence each other.

## Materials and Methods

This is a cross-sectional study based on Projecte Moviment (Castells-Sánchez et al., 2019) and is drawn from our previously published results (Castells-Sánchez et al., 2020). The study was carried out by the University of Barcelona in collaboration with Institut Universitari d'Investigació en Atenció Primària Jordi Gol and Hospital Germans Trias i Pujol. It was approved by the responsible ethics committees following the Declaration of Helsinki.

### Participants

One hundred and fifteen community dwelling healthy late-middle-aged adults were recruited from the Barcelona metropolitan area using multiple strategies (lists of volunteers from previous studies, advertisements in local media, presentations in local community organizations, etc.). They were 50–70 years old, were not cognitively impaired [Mini Mental State Examination, MMSE ≥ 24 (Blesa et al., 2001), and Montreal Cognitive Assessment 5-min, MoCA 5-min ≥ 6 (Wong et al., 2015)], had competency in Catalan or Spanish and had adequate sensory and motor skills. Participants were excluded from the study if they had a neurological diagnosis, psychiatric disease or Geriatric Depression Scale score >9 (Martínez et al., 2002), a history of drug abuse and alcoholism, consumed psychopharmacological drugs, history of chemotherapy, and had any contraindication to magnetic resonance imaging (MRI). As previously published (Castells-Sánchez et al., 2020), the sample consisted of Projecte Moviment baseline low-active participants (<2 h/week over the last 6 months) and 20 additional participants with a higher physical activity profile (≥5 h/week moderate PA or 2.5 h/week intense PA) in order to enlarge the interval of the PA and CRF levels and increase the sample size for a cross-sectional analysis. All participants were recruited, selected, and assessed following the same protocol during the same period of time. They were screened by phone and an on-site interview and signed an informed consent prior to the assessment.

### Assessments and Outcomes

All participants underwent a multimodal assessment organized into three appointments in 2 weeks in the following order: (1) Medical assessment and blood extraction (30 min), (2) Cognitive psychological health and physical activity assessment (2, 5 h), (3) MRI protocol (45 min). In order to control the effects of acute exercise, participants were advised not to exercise 8 h before all appointments. Assessments were conducted in clinical facilities: medical and cognitive assessments were carried out in two Primary Health Care Centers and MRI scans were performed in the Hospital Germans Trias i Pujol.

#### Physical Activity and Cardiorespiratory Fitness

Self-reported PA was evaluated by the Validated Spanish short version of Minnesota Leisure Time Physical Activity Questionnaire (VREM) (Ruiz et al., 2012). Participants reported frequency and duration of the following activities during the last month: sportive walking (walking in order to exercise), sport/dancing, gardening, climbing stairs, shopping, walking, and cleaning the house. We transformed hours per month expended in each category into units of metabolic equivalent of tasks (METs). This allowed us to estimate energy expenditure in Sportive PA (S-PA) adding up the METs spent in sportive walking and sportive/dancing activities.

We obtained estimated CRF applying the Rockport 1-Mile Walking Test which is a less invasive and valid method commonly used in healthy elderly population. Participants walked one mile on a treadmill (Technogym®, Italy) adjusting their speed in order to be as fast as possible without running. We registered time to complete the mile, heart rate, and average speed during the test once they finished. We estimated maximal aerobic capacity (VO_2_ max) using the linear regression developed by Kline et al. (1987).

#### Biomarkers

Blood extraction was performed between 8:00 and 9:00 a.m. following an overnight fast by nurses in the Primary Health Care Centers. All participants were instructed to not exercise 8 h before the blood test. Blood samples were obtained from the antecubital vein and collected in EDTA tubes for plasma analyses. Tubes were immediately transferred to the IGTP-HUGTP Biobank integrated in the Spanish National Biobanks Network of Instituto de Salud Carlos II (PT13/0010/0009) and Tumor Bank Network of Catalonia, and they were processed following standard operating procedures with the appropriate approval of the Ethical and Scientific Committees. Plasma aliquots were stored at −80°C.

As Level 1 outcomes, we obtained peripheral BDNF levels using an ELISA kit (Human Free BDNF Quantikine ELISA Kit; R&D Systems, Minnesota, USA). The rest of the molecular markers were selected according to the Projecte Moviment trial (Castells-Sánchez et al., 2019). TNF-α, ICAM-1, HGF, and SDF1-α levels were analyzed quantitatively using the corresponding ELISA immunoassay method (Human TNF-α Quantikine HS ELISA, Human ICAM-1/CD54 Allele-specific Quantikine ELISA Kit, Human HGF Quantikine ELISA Kit, Human CXCL12/SDF-1 alpha Quantikine ELISA Kit; R&D Systems, Minnesota, USA).

#### Neuroimaging

Structural MRI data was collected in a 3T Siemens Magnetom Verio Symo MR B17 (Siemens 243 Healthineers, Erlangen, Germany) for all participants (Castells-Sánchez et al., 2019). We acquired T1-weighted multi-planar reformat sequences (acquisition time: 5:26 min, voxel: 0.9 x 0.9 x 0.9 mm, TR/TE/TI: 1900/2.73/900 ms, flip angle: 9°, slices: 192; thickness: 0.9 mm) and an expert neuroradiologist visually checked them for artifacts or clinical brain conditions. All participants received a clinical report of the MRI.

Brain images were analyzed using MRICloud (https://mricloud.org/) (Mori et al., 2016), an online cloud-computing platform that includes a function to calculate gray and white matter brain volumes (Wu et al., 2016). It performs a fully automated parcellation of 287 volumes based on multiple atlases and fuses different algorithms (transformation algorithm, Large Deformation Diffeomorphic Metric Mapping (LDDMM), and the atlas label-fusion algorithm) (Christensen et al., 1997; Oishi et al., 2009; Wang et al., 2012) with a local search algorithm (Coupé et al., 2011). For our sample, we used atlas library version 10A, which includes 30 atlases from cognitively-normal individuals and individuals with cognitive impairment or dementia. For Level 2 outcomes, we selected relevant areas based on previous literature (Erickson et al., 2009, 2011; Verstynen et al., 2012) to perform analyses: ventricles, total WM and GM of the frontal lobe, dorsolateral prefrontal cortex, cingulate cortex, parietal lobe, precuneus, temporal lobe, and hippocampus. In accordance with other volumetric studies, we used the ANCOVA method to regress brain volumes on outcomes of interest. All volumes were normalized by head size by including intracranial volume (ICV) as a covariate. We calculated ICV summing volume of the brain tissue (Left Hemisphere + Right Hemisphere + Brainstem + Cerebellum) and CSF (Ventricles + Sulci).

#### Psychological Health and Daily Activity

Psychological health and daily activity of participants were assessed using self-reported questionnaires and the raw scores of each test were used as Level 3 outcomes. We applied GDS-15 (Martínez et al., 2002) for depressive symptoms, the Modified Version of Visual Analog Mood Scale (VAMS, Stern et al., 1997) for mood states and the Short Informant Questionnaire in Routine Evaluation-Outcome Measure (CORE-OM, Trujillo et al., 2016) to assess psychological distress in the domains of subjective well-being, problems/symptoms, general functioning, and risk. We also administered the Pittsburgh Sleep Quality Index (PSQI, Rico and Fernández, 1997) to evaluate the quality and patterns of sleep and the Short Informant Questionnaire on Cognitive Decline in the Elderly (S-IQCODE, Morales et al., 1992) as a measure of subjective cognitive performance in daily activities. We used the raw scores of each test as Level 3 outcomes.

#### Cognition and Demographic Data

We assessed cognitive function using an extensive neuropsychological battery which included standard tests selected for their psychometric qualities and high relevance in the area of study. These tests provided measures of multiple cognitive functions grouped following a theoretically-driven approach (Strauss and Spreen, 1998; Lezak et al., 2012) into five domains: (1) Executive (Inhibition, Flexibility, Fluency, Working Memory); (2) Visuospatial Function, (3) Language, (4) Memory (Verbal Memory, Visual Memory), (5) Attention-Speed (Attention, Speed). Extended details of the assessment are provided in the Supplementary Table 1. We obtained age, sex, and years of education, height and weight to calculate BMI, diagnoses of hypertension and diabetes as cardiovascular health variables and current medication for cardiovascular risk factors such as dyslipidemia, hypertension, and diabetes.

### Statistical Analyses

We used IBM SPSS Statistics for Windows, Version 24.0, for statistical analyses. Linear regression models were performed to examine the associations between S-PA and CRF with molecular (Level 1), brain volume (Level 2), and psychological (Level 3) outcomes. We stratified analyses by sex and included age and years of education as covariates. We also introduced BMI as a covariate for Level 1 and 2 analyses (Colbert et al., 2004; Bourassa and Sbarra, 2017) and ICV when using Level 2 outcomes. Since molecular outcomes in serum could be influenced by current cardiovascular risk factors medications, this variable was included as a dichotomous covariate in complementary analyses for Level 1 outcomes.

**Figure 1:**
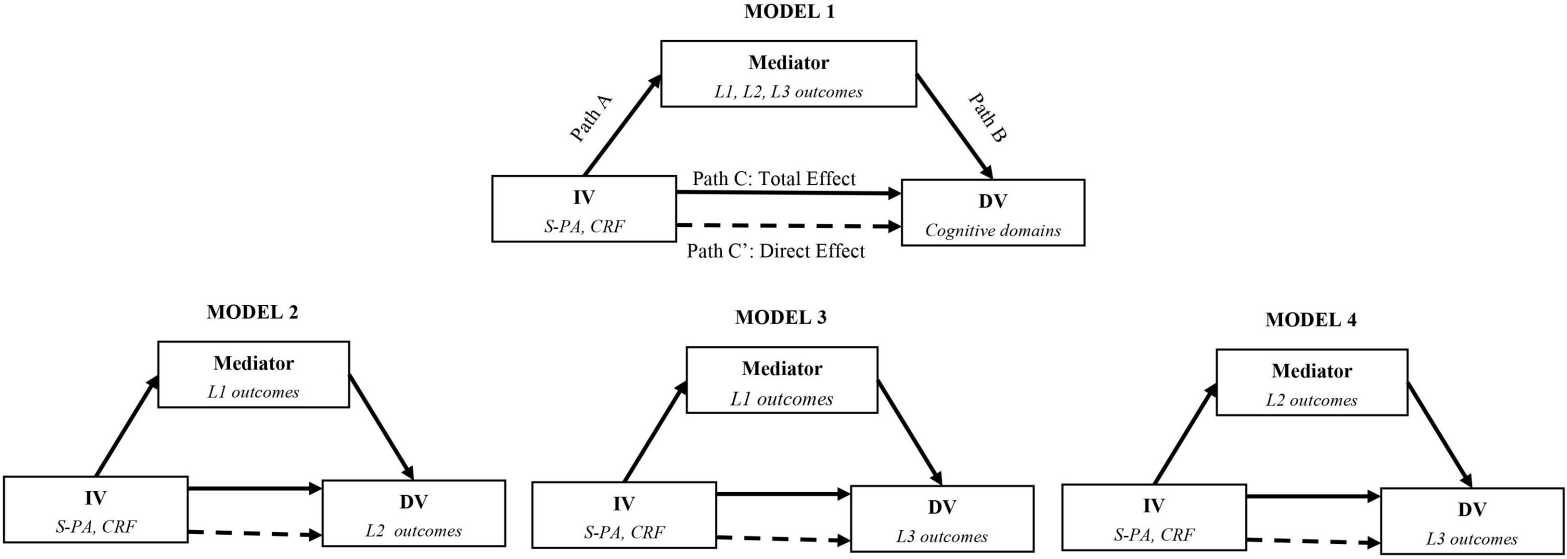
Mediating models.

We analyzed the potential mediating role of these outcomes in the relationship between S-PA and CRF with cognition in women and men separately (see Model 1 in Figure 1). Then, we performed exploratory mediation analyses addressing the potential pathways by which outcomes at Level 1 and 2 might influence markers at Level 2 and 3 (see Model 2, 3, and 4 in Figure 1). We applied mediation analyses using the PROCESS Macro (Figure 1). These analyses were computed with bias-corrected bootstrap or Monte Carlo 95% confidence intervals (CIs) based on 5,000 bootstrap samples. Significance of mediation was indicated if the confidence intervals (CIs) in Path AB did not overlap with 0 (Hayes, 2017).

## Results

### Participants

We recruited and assessed 115 healthy adults. The final sample consisted of 104 participants (age = 57.44 ± 5.36; 63% female; years of education = 13.35 ± 5.29; MMSE = 28.22 ± 1.45; BMI = 27.52 ± 4.91) from which we could obtain a valid measure of CRF. There were no significant differences in the demographic data between the 65 women and 39 men included in the sample except in years of education that is used as a covariate (see Table 1). Despite sex differences in S-PA and CRF levels, the correlations between S-PA and CRF in women (*r* = 0.551, *p* < 0.001) and men (*r* = 0.631, p < 0.001) were significant and comparable (see Table 1). There were no significant differences in age, years of education and MMSE scores between participants of Projecte Moviment and the 20 additional participants (see Supplementary Tables 2, 3).

**Table 1:** Demographic and physical activity variables.

	**Women**	**Men**	
**Demographic variables**	**Mean (SD)**	**Mean (SD)**	***t*****-Test/*****χ***^**2**^ **(*****p*****-Value)**
n	65	39	-
Age (years)	56.75 (4.96)	58.59 (5.86)	1.64 (0.106)
Education (years)	12.46 (4.97)	14.82 (5.55)	2.18 (0.032)
MMSE (/30)	28.15 (1.41)	28.33 (1.53)	0.60 (0.552)
BMI (kg/m^2^)	26.99 (4.53)	28.40 (5.44)	−1.43 (0.157)
Hypertension (n)	12	10	3.46 (0.063)
Diabetes (n)	7	4	3.59 (0.058)
**Physical activity variables**	**Mean (SD)**	**Mean (SD)**	***t*****-Test (*****p*****-Value)**
S-PA (METs)	2055.35 (4395.04)	6011.35 (9939.24)	−2.35 (0.023)
CRF (ml/kg*min)	25.07 (12.31)	33.86 (12.92)	−3.46 (0.001)
1-Mile heart rate (bpm)^a^	90.27 (16.25)	93.97 (22.02)	1.11 (0.269)
1-Mile walking time (min)^a^	18.31 (3.38)	16.21 (3.48)	−2.17 (0.033)

*MMSE, Mini-Mental State Examination (Blesa et al., 2001); S-PA, Sportive Physical Activity; CRF, Cardiorespiratory fitness; METs, Metabolic equivalent*.

a *Variables obtained at the completion of the Rockport 1-Mile Walking Test included in the CRF estimation model*.

### Associations Between Physical Activity, CRF, and Mechanisms at Level 1, 2, and 3

#### Level 1: Physical Activity, CRF, and Molecular Biomarkers

Linear regression models examining the relationship between S-PA and CRF with Level 1 biomarkers in women and men separately are in Table 2. Engaging in greater amounts of S-PA was significantly associated with reduced levels of TNF-α in women. There were not any significant associations between S-PA and molecular markers in men. In contrast, higher CRF levels were associated with lower TNF-α and HGF levels in men. There were not significant relationship between CRF and molecular markers in women. Extended details for each model are included in Supplementary Table 4.1. Besides, similar results were obtained for these same regression linear models when accounting for the potential influence of cardiovascular risk factors medications as a covariate (see Supplementary Table 5).

**Table 2:** Linear regression models in women and men: relationship between physical activity variables and molecular biomarkers.

**Molecular biomarkers**	**Women**		**Men**	
	**S-PA** ** β (*p*-value)**	**CRF** ** β (*p*-value)**	**S-PA** ** β (*p*-value)**	**CRF** ** β (*p*-value)**
BDNF (pg/ml)	−0.04 (0.797)	−0.09 (0.646)	−0.09 (0.642)	−0.32 (0.224)
TNF-α (pg/ml)	−0.39 (0.007)^**^	−0.19 (0.288)	−0.19 (0.304)	−0.65 (0.017)^*^
HGF (pg/ml)	−0.12 (0.398)	−0.25 (0.143)	−0.23 (0.152)	−0.58 (0.018)^*^
ICAM-1 (ng/ml)	−0.12 (0.473)	−0.32 (0.082)	−0.15 (0.423)	−0.07 (0.820)
SDF1-α (pg/ml)	0.06 (0.695)	0.27 (0.132)	0.06 (0.781)	−0.07 (0.821)

*S-PA, Sportive Physical Activity; CRF, Cardiorespiratory Fitness; β, standardized beta*.

*Covariates: age, years of education, BMI*.

S-PA is measured in METs units and CRF in ml/kg*min.

* p < 0.05;

** *p < 0.01*.

#### Level 2: Physical Activity, CRF, and Brain Volumes

Linear regression models examining the relationship between S-PA and CRF with brain volumes, Level 2 outcomes, in women and men separately are described in Table 3. Engaging in greater amounts of S-PA were positively correlated with the volume of the dorsolateral prefrontal cortex in women and with temporal lobe volume in both women and men. There was also a relationship between S-PA and precuneus volume in men. Greater levels of CRF were associated with larger precuneus and temporal lobes and with smaller ventricles in men. There was also a correlation between CRF and frontal and parietal lobe volumes in men. In women, there were no significant relationships between CRF and brain volumes. Extended details for each model are included in Supplementary Table 4.2.

**Table 3:** Linear regression models in women and men: relationship between physical activity variables and brain volumes.

**Brain volumes (mm^**3**^)**	**Women**		**Men**	
	**S-PA** ** β (*p*-value)**	**CRF** ** β (*p*-value)**	**S-PA** ** β (*p*-value)**	**CRF** ** β (*p*-value)**
Ventricles	0.23 (0.104)	0.08 (0.642)	−0.28 (0.113)	−0.69 (0.002)^**^
Total white matter	−0.02 (0.739)	0.06 (0.442)	−0.07 (0.530)	−0.07 (.616)
Frontal lobe	−0.03 (0.632)	0.02 (0.778)	0.11 (0.260)	0.23 (0.074)
Dorsolateral prefrontal cortex	0.22 (0.015)*	0.19 (0.074)	0.06 (0.632)	−0.09 (0.618)
Cingulate cortex	0.12 (0.183)	−0.02 (0.856)	0.18 (0.134)	0.05 (0.754)
Parietal lobe	−0.00 (0.965)	−0.07 (0.377)	0.09 (0.279)	0.20 (0.051)
Precuneus	−0.07 (0.545)	−0.12 (0.404)	0.28 (0.054)	0.46 (0.014)^*^
Temporal lobe	0.14 (0.041)^*^	0.11 (0.191)	0.20 (0.033)^*^	0.25 (0.048)^*^
Hippocampus	0.07 (0.567)	−0.02 (0.899)	−0.07 (0.694)	−0.06 (0.779)

*S-PA, Sportive Physical Activity; CRF, Cardiorespiratory Fitness; β, standardized beta*.

*Covariates: age, years of education, ICV, BMI*.

S-PA is measured in METs units and CRF in ml/kg*min.

* p < 0.05;

** *p < 0.01*.

#### Level 3: Physical Activity, CRF, and Psychological Health and Daily Activity

Linear regression models examining the relationship between S-PA and CRF with behavioral outcomes at Level 3 in women and men separately are in Table 4. There were no significant associations between S-PA and any of the scores in women and men. However, when analyzing the subscales, we found that in men, higher levels of S-PA were related to better subjective sleep efficiency (β = 0.41, *p* = 0.008) and fewer sleep disturbances (β = −0.43, *p* = 0.008) in the Pittsburgh Sleep Quality Index and higher scores of well-being (β = −0.31, *p* = 0.065) in the corresponding subscale of the CORE-OM test. In addition, in men, higher CRF was significantly associated with fewer depressive symptoms measured by the GDS. There was also a moderate negative relationship between levels of CRF and VAMS scores in men. Extended details for each model are included in Supplementary Table 4.3.

**Table 4:** Linear regression models in women and men: relationship between physical activity variables and behavior outcomes.

**Psychological status and daily activity**	**Women**		**Men**	
	**S-PA** ** β (*p*-value)**	**CRF** ** β (*p*-value)**	**S-PA** ** β (*p*-value)**	**CRF** ** β (*p*-value)**
GDS	−0.16 (0.207)	−0.05 (0.729)	−0.23 (0.174)	−0.39 (0.021)^*^
VAMS	−0.10 (0.446)	−0.26 (0.070)	−0.06 (0.717)	−0.30 (0.055)
S-IQCODE	−0.02 (0.875)	0.19 (0.193)	−0.14 (0.403)	−0.16 (0.354)
PSQI	−0.11 (0.416)	−0.04 (0.784)	−0.13 (0.445)	−0.19 (0.286)
Total CORE-OM	−0.19 (0.138)	−0.17 (0.238)	−0.15 (0.378)	−0.10 (0.547)

*S-PA, Sportive Physical Activity; CRF, Cardiorespiratory Fitness; β, standardized beta; GDS, Geriatric Depression Scale (Martínez et al., 2002); VAMS, Visual Analog Mood Scale (Stern et al., 1997); S-IQCODE, Short Informant Questionnaire on Cognitive Decline in the Elderly (Morales et al., 1992); PSQI, Pittsburgh Sleep Quality Index (Rico and Fernández, 1997); CORE-OM, Short Informant Questionnaire in Routine Evaluation-Outcome Measure (Trujillo et al., 2016)*.

*Covariates: age, years of education*.

S-PA is measured in METs units and CRF in ml/kg*min.

* *p < 0.05*.

### Mediating Effects

#### Model 1

**Figure 2:**
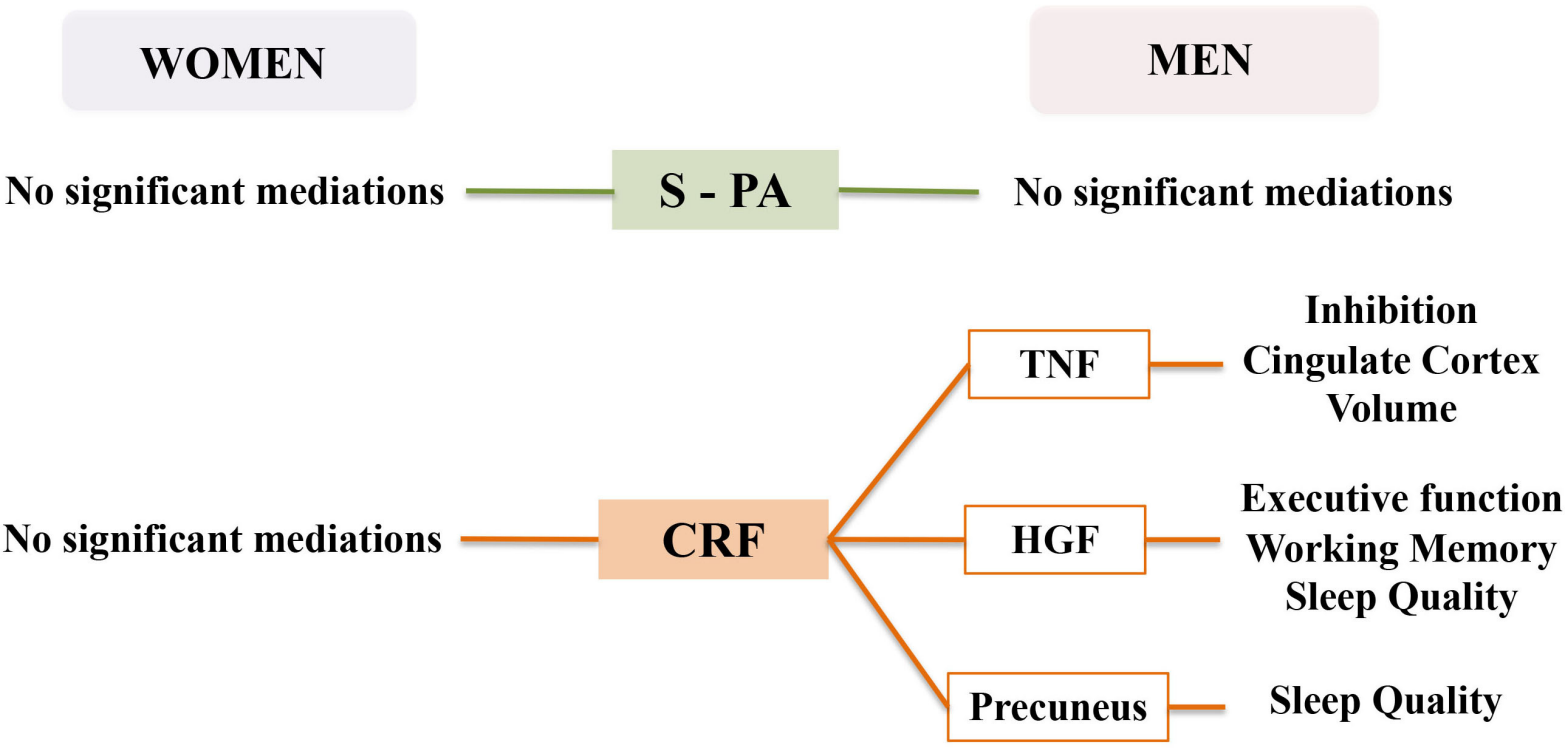
Mediation results of Models 1, 2, 3, and 4 in women and men.

Model 1 (see Figure 1) tested the mediating effect of each molecular, brain volume and behavioral outcome in the relationship between S-PA and CRF with the assessed cognitive functions in women and men. In the relationship between S-PA and cognitive domains, none of the outcomes at Level 1, two or three showed significant indirect effects in the mediation analyses in women or men. When CRF was the predictor, we found statistically significant indirect effects for HGF in the CRF-executive function (Path AB = β = 0.29, SE = 0.17, 95% CI: 0.02, 0.70) and in the CRF—working memory (Path AB = β = 0.25, SE = 0.15, 95% CI: 0.02, 0.59) relationships in men. Indirect effects were also significant when TNF-α was the mediator in the association between CRF and inhibition (Path AB = β = 0.31, SE = 0.19, 95% CI: 0.04, 0.77) only in men. There were no significant indirect effects for any outcome in the CRF-cognition relationship in women (see Figure 2).

#### Model 2

Model 2 (see Figure 1) examined the mediating effects of molecular outcomes in the relationship between S-PA and CRF with brain volumes in women and men. In the association between S-PA and brain volumes, none of the molecular outcomes showed significant mediating effect in women or men. In the mediation analyses with CRF as a predictor, we found statistically significant indirect effects for TNF-α in the relationship between CRF and cingulate cortex volume (Path AB = β =-0.26, SE = 0.14, 95% CI: −0.58, −0.03) only in men. We did not find any significant mediating effects for any molecular outcome in the CRF-brain volume association in women (see Figure 2).

#### Model 3

Model 3 (see Figure 1) assessed the mediating effects of molecular outcomes in the relationship between S-PA and CRF with psychological health and daily activity. In the relationship between S-PA and behavioral outcomes, none of the molecular outcomes showed significant indirect effects in women or men. When CRF was the predictor, we found statistically significant indirect effects for HGF in the relationship between CRF and the subscale of subjective sleep quality of the Pittsburgh Sleep Quality Index (Path AB = β = −0.21, SE = 0.13, 95% CI: −0.53, −0.03) in men. There were no significant mediating effects for any molecular outcome in the CRF-behavioral relationship in women (see Figure 2).

#### Model 4

Model 4 (see Figure 1) tested the mediating effects of brain volume outcomes in the relationship between S-PA and CRF with psychological health and daily activity. In the association between S-PA and behavioral outcomes, none of the brain volume outcomes showed significant mediating effects in women or men. In the mediation analyses with CRF as a predictor, we found statistically significant indirect effects of precuneus volume in the relationship between CRF and a subscale of subjective sleep quality from the Pittsburgh Sleep Quality Index (Path AB = β = −0.26, SE = 0.17, 95% CI: −0.65, −0.00) in men. We did not find any significant mediating effects for any brain volume outcome in the CRF-behavioral association in women (see Figure 2).

## Discussion

To our knowledge, we are the first to describe the relationship between physical activity outcomes and three different levels of potential mechanisms–molecular, brain volume and behavioral–in healthy late-middle-aged women and men. Moreover, we analyzed these potential mediators in the relationship between exercise and CRF with cognition and between themselves stratifying results by sex.

At the molecular level, Level 1, our results support previous evidence (Sallam and Laher, 2016) suggesting that physical activity might be related to reduced levels of inflammation. However, despite that self-reported exercise and CRF measures were highly correlated, we found sex differences when examining these outcomes. In particular, we found that reduced TNF-α levels were related to greater energy expenditure in sportive physical activity only in women and to higher CRF levels only in men. Men with higher CRF also showed reduced levels of HGF, independently of BMI. Both biomarkers are related to pro-inflammatory processes, obesity, and insulin resistance and, therefore, reduced levels might induce a neuroprotective effect (Kiliaan et al., 2014). Nevertheless, we can not discard that these results could be related to other processes besides inflammation given the pleiotropic nature of these markers. Interestingly, when analyzing the mediating role of molecular outcomes in the physical activity-cognition association, we found that the reduced levels of these inflammation-related markers might be an important mediator of the brain and cognitive CRF-related benefits. In men, HGF was a significant mediator in the association between CRF and executive function, working memory, and sleep quality and TNF-α was a significant mediator in the relationship between CRF and inhibition. Interestingly, those men with higher CRF also showed that TNF-α was a significant mediator of the cingulate cortex volume, which is functionally involved in executive function processes such as inhibition (Huang et al., 2020). This fact highlights the potential role of reduced neuroinflammation as a result of exercise and enhanced CRF to promote greater executive functions.

Our findings at brain volume level, Level 2, are consistent with previous findings that physical activity has been consistently associated with the maintenance of brain volume and with less atrophy across the lifespan (Erickson et al., 2014). One of our relevant results is that frequent exercise was related with greater temporal lobe volume in women and men, which is a key structure for memory and it deteriorates with aging. Moreover, consistent with Barha et al. (2019), women that expended more energy in sportive physical activities had greater dorsolateral prefrontal cortex volume, which is involved in supporting executive functions, especially working memory. In other studies greater CRF levels have been linked to greater brain volumes (Raichlen et al., 2019; Wittfeld et al., 2020). Interestingly, in our study, higher CRF levels were associated with less ventricular volume and higher volumes of temporal and parietal lobes, specifically of the precuneus, in men but not in women. Despite previously published evidence for the mediating effects of brain volume in the relationship between physical activity outcomes and cognition, such as the mediating role of the hippocampus in the association between CRF and spatial memory (Erickson et al., 2009), we found that physical activity and fitness were associated with larger volumes, but that this was unrelated to cognition.

Previous literature about Level 3 outcomes reported that active lifestyles such as exercise are also related with mood (Fox et al., 2007; Willis et al., 2018) and sleep patterns (Kline et al., 2013) in older adults. Curiously, we found similar results, but this was significant only in men. Men performing more exercise reported higher sleep efficiency and fewer sleep disturbances and those with higher levels of CRF presented fewer depressive symptoms and better general mood. In mediation analyses, and in accordance with previous papers relating diminished precuneus with insomnia (Grau-Rivera et al., 2020) and sleep restriction (Long et al., 2020), we found that greater volume of the precuneus in men mediated the higher CRF-better subjective sleep quality association. Surprisingly, in our sample, physical activity was not related with these behavioral outcomes in women. Based on previous papers, we hypothesize that mood (Wharton et al., 2012) and sleep (Baker et al., 2020) patterns in women could be influenced by other parameters, such as hormonal changes.

Our results are consistent with previous findings about exercise on health outcomes as we described above. Nevertheless, we not only add support to the multi-level benefits of regular exercise but also highlight the sex differences in the role of each physical activity outcome as we stratified results by sex. CRF was an outcome highly related to cognition and to the molecular, brain, and psychological cascade of changes in men but not in women. However, benefits of exercise at the molecular level including reduced TNF-α levels and brain volumes –dorsolateral prefrontal cortex and temporal lobe, were also observed in women but were linked to the amount of self-reported energy expended in sportive activities during last month and not to CRF. These results could be related to sex differences in the musculoskeletal and cardiovascular systems and their adaptations in response to exercise (Barha and Liu-Ambrose, 2018; Ansdell et al., 2020). Current literature suggests that women experience less metabolic stress for the same amount of exercise and, in turn, lesser adaptative response (e.g., less increase in CRF levels). Moreover, evidence suggests that the integrative response to exercise might be mediated by an oestrogenic effect in females. Therefore, differences in sex hormones that act as neurosteroids and interact with molecular growth factors, brain structures and cognition in a different manner for women and men could explain part of this variability (Ansdell et al., 2020). Besides, this is in accordance with recent bibliography stating sex differences in the association of exercise with brain and cognitive outcomes (Lindwall et al., 2008; Varma et al., 2015, 2016; Barha et al., 2019; Dimech et al., 2019). Another potential explanation could be a dose-effect bias related to the described differences in the amount of physical activity performed by women and men. Although, this fact could be a source of variance, sex differences in the distribution of SPA and CRF observed in our sample are consistent with previously published literature reporting that men are more physically active (Al-Mallah et al., 2016). Additionally, there is evidence reporting no significant differences between PA levels but showing sex differences in CRF levels and significant associations between CRF and functional brain outcomes only in men (Dimech et al., 2019). Therefore, future studies should address the influence of dose by sex in sex-balanced samples.

It must be acknowledged that our results are based on a cross-sectional design which allows us to describe statistical relationships but not causal conclusions about the neuroprotective effects of exercise. Moreover, further studies should address these aims in samples with different physical activity profiles, age groups, sex-balanced groups, and a non-estimated measure of CRF. Including Heart Rate Reserve to estimate intensity/exertion during the test could provide further information about the physical activity status of participants and inform about its role in relation with physiological mechanisms. We stress the need to stratify results by sex and study the role of the hormonal profile in the molecular cascade that might explain the benefits of exercise to brain health.

## Conclusion

From a clinical perspective, we showed that exercise might be related to reduced levels of inflammatory markers and increased brain volume in areas commonly deteriorating in aging. Regular exercise is also associated to better psychological and sleep health in men.

Our results contribute to the field of research adding evidence about the mediating effects of molecular biomarkers capable to modulate the immune system in the relationship between CRF and cognition in men. Moreover, our results suggest sex differences in the association between physical activity outcomes and molecular, brain and psychological outcomes. This might be related to sex differences in the musculoskeletal and cardiovascular adaptations after exercise as well as sex differences in the hormonal profile. Future studies should address the interaction of the hormonal profile with the molecular and brain measures commonly studied in cross-sectional designs and RCTs.

## Data Availability Statement

The raw data supporting the conclusions of this article will be made available by the authors, without undue reservation.

## Ethics Statement

The studies involving human participants were reviewed and approved by Bioethics Commission of the University of Barcelona (IRB00003099) and Clinical Research Ethics Committee of IDIAP Jordi Gol (P16/181). The patients/participants provided their written informed consent to participate in this study.

## Author Contributions

AC-S and FR-C participated in the study concept and design, acquisition, analyses, and interpretation of data as well as in the elaboration of the manuscript. RD-A contributed processing the neuroimaging data. NL-V collaborated in the acquisition of the data. RD-A, NL-V, AKS, PT-M, GP, PM-A, AH-T, SD, MV, and KE critically reviewed the content of the article. MM conceptualized the study, contributed to the study design and the implementation as Principal Investigator and supervised all procedures, and the elaboration of the manuscript. All authors contributed to the article and approved the submitted version.

## Conflict of Interest

The authors declare that the research was conducted in the absence of any commercial or financial relationships that could be construed as a potential conflict of interest.
