# Supplementary material for: Exercise and Fitness Neuroprotective Effects: Molecular, Brain Volume and Psychological Correlates and Their Mediating Role in Healthy Late-Middle-Aged Women and Men
Source: Front Aging Neurosci. 2021 Mar 8;13:615247. doi: 10.3389/fnagi.2021.615247 (PMC7989549; doi:10.3389/fnagi.2021.615247)
Supplement: Supplementary file 1 [file Table_1.DOCX]

| **Table 1. Cognitive outcomes: variables and measures** | | | |
| --- | --- | --- | --- |
| **Composites 1^st^ Level** | **Composites 2^nd^ Level** | **Tests - Subtest** | **Measure** |
| Executive Function | Flexibility | TMT B -A | Z score |
|  | Fluency | Letter fluency | Z score |
|  |  | Category fluency | Z score |
|  | Inhibition | Stroop - Interference | Z score |
|  | Working Memory | WAIS III - Backward Span | Z score |
| Visuospatial Function | Visuospatial Function | ROCF - Copy Accuracy | Z score |
| Language | Language | BNT (15 items) | Z score |
| Attention - Speed | Attention | WAIS III - Forward Span | Z score |
|  |  | WAIS III - Digit Symbol Coding | Z score |
|  |  | WAIS-III - Symbol Search | Z score |
|  | Speed | TMT - A | Z score |
|  |  | ROCF - Copy Time | Z score |
| Memory | Visual Memory | ROCF - Memory Accuracy | Z score |
|  | Verbal Memory | RAVLT - Total Learning | Z score |
|  |  | RAVLT - Recall II | Z score |
| *Note: TMT = Trail Making Test; Verbal Fluency Tests; Stroop Test; WAIS-III = Wechsler Adult Intelligence Scale; ROCF = Rey-Osterrieth Complex Figure; BNT = Boston Naming Test; RAVLT = Rey Auditory Verbal Learning Test* | | | |
