# Supplementary material for: Exercise and Fitness Neuroprotective Effects: Molecular, Brain Volume and Psychological Correlates and Their Mediating Role in Healthy Late-Middle-Aged Women and Men
Source: Front Aging Neurosci. 2021 Mar 8;13:615247. doi: 10.3389/fnagi.2021.615247 (PMC7989549; doi:10.3389/fnagi.2021.615247)
Supplement: Supplementary file 2 [file Table_2.docx]

| Table 2. Demographic variables Projecte Moviment sample vs. extra participants | | | |
| --- | --- | --- | --- |
|  | **Projecte Moviment sample**  **Mean (SD)** | **Extra participants**  **Mean (SD)** | **t-Test, *p* Value** |
| n total / n female | 84/56 | 20/9 | - |
| Age (years) | 57.56 (5.46) | 56.95 (5.04) | 0.46, .650 |
| Education (years) | 12.95 (5.38) | 15.00 (4.67) | -1.57, .121 |
| MMSE (/30) | 28.12 (1.36) | 28.65 (1.73) | -1.48, .141 |
| *Note: MMSE = Mini-Mental State Examination* | | | |
