# Supplementary material for: Exercise and Fitness Neuroprotective Effects: Molecular, Brain Volume and Psychological Correlates and Their Mediating Role in Healthy Late-Middle-Aged Women and Men
Source: Front Aging Neurosci. 2021 Mar 8;13:615247. doi: 10.3389/fnagi.2021.615247 (PMC7989549; doi:10.3389/fnagi.2021.615247)
Supplement: Supplementary file 3 [file Table_3.docx]

| **Table 3. PA and CRF outcomes in Projecte Moviment sample and extra participants.** | | | | | |
| --- | --- | --- | --- | --- | --- |
|  | **Projecte Moviment sample** | | | **Extra participants** | |
|  | **Mean (SD)** | **[Min – Max]** | **Mean (SD)** | | **[Min – Max]** |
| S-PA | 497.48 (7723.75) | [0 - 3066] | 16312.60 (8232.39) | | [4524 - 36952] |
| CRF | 24.42 (11.14) | [0.07 - 44.70] | 44.91 (6.67) | | [31.16 - 58.64] |
| *Note: S-PA = Sportive Physical Activity; CRF = Cardiorespiratory Fitness.* | | | | | |
