# Supplementary material for: Exercise and Fitness Neuroprotective Effects: Molecular, Brain Volume and Psychological Correlates and Their Mediating Role in Healthy Late-Middle-Aged Women and Men
Source: Front Aging Neurosci. 2021 Mar 8;13:615247. doi: 10.3389/fnagi.2021.615247 (PMC7989549; doi:10.3389/fnagi.2021.615247)
Supplement: Supplementary file 4 [file Table_4.docx]

| Table 4.1. Data regarding linear regression models for the relationship between physical activity outcomes and molecular biomarkers in women and men. | | | | |
| --- | --- | --- | --- | --- |
| Molecular biomarkers | **WOMEN** | | **MEN** | |
|  | **S-PA**  *R ^2^ ,(df)F, p-value* | **CRF**  *R ^2^ ,(df)F, p-value* | **S-PA**  *R ^2^ ,(df)F, p-value* | **CRF**  *R ^2^ ,(df)F, p-value* |
| BDNF (pg/ml) | .07, (4,51)1.02, .408 | .08, (4,51)1.06, .388 | .13, (4,32)1.16, .348 | .16, (4,32)1.53, .216 |
| TNF-α (pg/ml) | .27, (4,50)4.65, .003 | .17, (4,50)2.61, .046 | .22, (4,31)2.18, .094 | .33, (4,31)3.81, .012 |
| HGF (pg/ml) | .19, (4,50)2.98, .028 | .22, (4,50)3.43, .015 | .40, (4.31)5.11, .003 | .46, (4,31)6.70, .001 |
| ICAM-1 (ng/ml) | .04, (4,50)0.47, .759 | .08, (4,50)1.14, .348 | .16, (4,31)1.47, .234 | .14, (4,31)1.30, .292 |
| SDF-1a (pg/ml) | .10, (4,50)1.32, .277 | .13, (4,50)1.92, .122 | .09, (4,31)0.78, .550 | .09, (4,31)0.77, .554 |
| *Note: S-PA=Sportive Physical Activity; CRF=Cardiorespiratory Fitness; β=standardized beta*  *Covariates: age, years of education, BMI.*  *S-PA is measured in METs units and CRF in ml/kg*min* | | | | |

| **Table 4.2.** Data regarding linear regression models for the relationship between physical activity variables and brain volumes in women and men. | | | | |
| --- | --- | --- | --- | --- |
| **Brain Volumes**  (mm^3^) | **WOMEN** | | **MEN** | |
|  | **S-PA**  *R ^2^ ,(df)F, p-value* | **CRF**  *R ^2^ ,(df)F, p-value* | **S-PA**  *R ^2^ ,(df)F, p-value* | **CRF**  *R ^2^ ,(df)F, p-value )* |
| Ventricles | .30, (5,52)4.41, .002 | .26 (5,52)3.73, .006 | .49, (5,29)5.48, .001 | .61, (5,29)8.89, <.001 |
| Total White Matter | .83, (5,52)49.73, <.001 | .82, (5,52)50.30, <.001 | .82, (5,29)25.92, <.001 | .82, (5,29)25.77, <.001 |
| Frontal Lobe | .86, (5,52)63.06, <.001 | .86, (5,52)62.84, <.001 | .84, (5,29)29.52, <.001 | .85, (5,29)31.99, <.001 |
| Dorsolateral Prefrontal Cortex | .72, (5,52)26.17, <.001 | .70(5,52)24.31, <.001 | .71, (5,29)14.10, <.001 | .71, (5,29)14.11, <.001 |
| Cingulate Cortex | .71, (5,52)25.84, <.001 | .70(5,52)24.63, <.001 | .76, (5,29)18.74, <.001 | .75, (5,29)16.96, <.001 |
| Parietal Lobe | .83, (5,52)49.22, <.001 | .83, (5,52)50.13, <.001 | .89, (5,29)48.87, <.001 | .90, (5,29)54.16, <.001 |
| Precuneus | .48, (5,52)9.43, <.001 | .48, (5,52)9.55, <.001 | .66, (5,29)11.12, <.001 | .69, (5,29)12.59, <.001 |
| Temporal Lobe | .83, (5,52)51.66, <.001 | .82, (5,52)48.77, <.001 | .86, (5,29)34.56, <.001 | .85, (5,29)33.65, <.001 |
| Hippocampus | .44, (5,52)8.21, <.001 | .44, (5,52)8.10, <.001 | .47, (5,29)5.21, .002 | .47, (5,29)5.18, .002 |
| *Note: S-PA=Sportive Physical Activity; CRF=Cardiorespiratory Fitness; β=standardized beta*  *Covariates: age, years of education, ICV, BMI*  *S-PA is measured in METs units and CRF in ml/kg*min* | | | | |

| **Table 4.3.** Data regarding linear regression models for the relationship between physical activity variables and behavior outcomes in women and men. | | | | |
| --- | --- | --- | --- | --- |
| **Psychological Status & Daily Activity** | **WOMEN** | | **MEN** | |
|  | **S-PA**  *R ^2^ ,(df)F, p-value* | **CRF**  *R ^2^ ,(df)F, p-value* | **S-PA**  *R ^2^ ,(df)F, p-value* | **CRF**  *R ^2^ ,(df)F, p-value )* |
| GDS | .09, (3,60)2.00, .123 | .07, (3,60)1.46, .234 | .07, (3,35)0.84, .482 | .16, (3,35)2.15, .111 |
| VAMS | .02, (3,61)0.39, .761 | .06, (3,61)1.34, .270 | .18, (3,35)2.62, .066 | .26, (3,35)4.16, .013 |
| S-IQCODE | .00, (3,61)0.05, .986 | .03, (3,61)0.62,.606 | .06, (3,35)0.67, .574 | .06, (3,35)0.73, .541 |
| PSQI | .01, (3,61)0.25, .683 | .00, (3,61)0.05, .986 | .03, (3,35)0.38, .768 | .05, (3,35)0.57, .636 |
| Total CORE-OM | .12, (3,61)2.69, .054 | .11, (3,61)2.38, .078 | .06, (3,35)0.72, 545 | .05, (3,35)0.58, .636 |
| *Note: S-PA=Sportive Physical Activity; CRF=Cardiorespiratory Fitness; GDS= Geriatric Depression Scale (Martínez et al., 2002); VAMS=Visual Analog Mood Scale (Stern et al., 1997); S-IQCODE= Short Informant Questionnaire on Cognitive Decline in the Elderly (Morales et al., 1992); PSQI= Pittsburgh Sleep Quality Index (Rico & Fernández, 1997); CORE-OM=Short Informant Questionnaire in Routine Evaluation-Outcome Measure (Trujillo et al., 2016)*  *Covariates: age, years of education*  *S-PA is measured in METs units and CRF in ml/kg*min* | | | | |
