# Supplementary material for: Exercise and Fitness Neuroprotective Effects: Molecular, Brain Volume and Psychological Correlates and Their Mediating Role in Healthy Late-Middle-Aged Women and Men
Source: Front Aging Neurosci. 2021 Mar 8;13:615247. doi: 10.3389/fnagi.2021.615247 (PMC7989549; doi:10.3389/fnagi.2021.615247)
Supplement: Supplementary file 5 [file Table_5.docx]

| Table 5. Linear regression models in women and men: relationship between physical activity outcomes and molecular biomarkers | | | | |
| --- | --- | --- | --- | --- |
| Molecular biomarkers | **WOMEN** | | **MEN** | |
|  | **S-PA**  *β (p-value)* | **CRF**  *β (p-value)* | **S-PA**  *β (p-value)* | **CRF**  *β (p-value)* |
| BDNF (pg/ml) | -.03 (.844) | -.07 (.720) | -.13 (.484) | -.31 (.245) |
| TNF-α (pg/ml) | -.40 (.006)** | -.17 (.326) | -.26 (.178) | -.71 (.009)* |
| HGF (pg/ml) | -.15 (.318) | -.25 (.148) | -.27 (.118) | -.61 (.015)* |
| ICAM (ng/ml) | -.12 (.464) | -.33 (.087) | -.12 (.545) | -.03 (.917) |
| SDF1-α (pg/ml) | .07 (.673) | .30 (.096) | .12 (.552) | -.02 (.956) |
| *Note: S-PA=Sportive Physical Activity; CRF=Cardiorespiratory Fitness; β=standardized beta*  *Covariates: age, years of education, BMI, cardiovascular risk factors medication*  *S-PA is measured in METs units and CRF in ml/kg*min*  ** p <.05; ** p <.01.* | | | | |
